# A257 PANCREATIC CANCER TREATMENT AND END OF LIFE OUTCOMES: A POPULATION BASED COHORT STUDY

**DOI:** 10.1093/jcag/gwab049.256

**Published:** 2022-02-21

**Authors:** R Khan, M Salim, P Tanuseputro, A Hsu, N Coburn, R Talarico, P James

**Affiliations:** 1 Department of Medicine, University of Toronto, Toronto, ON, Canada; 2 University Health Network, Toronto, ON, Canada; 3 Department of Medicine in Ottawa, Ottawa, ON, Canada; 4 University of Ottawa Department of Family Medicine, Ottawa, ON, Canada; 5 University of Toronto Division of General Surgery, Toronto, ON, Canada; 6 ICES (Formerly Institute for Clinical and Evaluative Sciences), Ottawa, ON, Canada

## Abstract

**Background:**

Patients with pancreatic cancer face challenging decisions regarding treatment choices following their diagnosis and often lack data on end-of-life (EOL) outcomes. Without the available information, older patients may be undertreated, dying earlier than they would have with treatment, while others may be overtreated and exposed to aggressive measures with harmful side effects.

**Aims:**

To describe survival and EOL outcomes among pancreatic cancer patients based on index cancer treatment, disease stage, and patient characteristics.

**Methods:**

We conducted a population based cohort study in Ontario, Canada of patients who died from April 2010 to December 2017 and were diagnosed with pancreatic cancer prior to death. We used administrative databases to collect data on demographics, baseline health status, treatments, and outcomes. The primary exposure was index cancer treatment (no treatment, radiation, chemotherapy alone, surgery alone, and surgery with chemotherapy). The primary outcomes were mortality, health care encounters per 30 days in the last six months of life, and palliative care visits per 30 days within the last six months of life. Secondary outcomes were location of death (institution vs. community), hospitalization within the last 30 days of life, and receipt of chemotherapy within the last 30 days of life. We estimated the association between the exposure and outcomes using multivariable models, adjusting for demographics, comorbidities, and cancer stage. Hazard ratios, adjusted mean differences, and odds ratios were reported with 95% confidence intervals.

**Results:**

Our cohort included 9950 adults with a median age at diagnosis of 78. 56% received no index treatment, 5% underwent radiation, 27% underwent chemotherapy alone, 7% underwent surgery alone, and 6% underwent surgery and chemotherapy. In the multivariable regression (**Table** and **Figure**), radiation, chemotherapy alone, surgery alone, and surgery with chemotherapy were all associated with decreased mortality and fewer healthcare encounters. All groups except radiation were associated with fewer palliative care visits. All treatment groups were associated with lower odds of institutional death and hospitalization within the last 30 days of life, and higher odds of chemotherapy within the last 30 days of life.

**Conclusions:**

Our data, the first to provide EOL outcome estimates based on index cancer treatment, can help patients make initial treatment decisions after a diagnosis of pancreatic cancer.

Multivariable regression analyses predicting primary and secondary outcomes

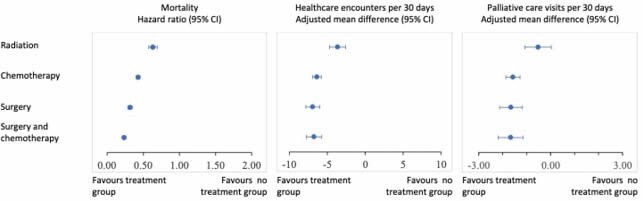

Association between index cancer treatment and primary outcomes.

**Funding Agencies:**

CIHR

**Table:** 

Treatment group	Mortality	Healthcare encounters per 30 days	Palliative care visits per 30 days	Institutional death	Hospitalization within last 30 days of life	Chemotherapy within last 30 days of life
No treatment	Referent	Referent	Referent	Referent	Referent	Referent
Radiation	0.63 (0.57 to 0.70)	-3.64 (-4.71 to -2.58)	-0.52 (-1.07 to 0.03)	0.79 (0.66 to 0.97)	0.66 (0.54 to 0.80)	4.13 (2.06 to 8.27)
Chemotherapy alone	0.43 (0.41 to 0.45)	-6.35 (-6.92 to -5.78)	-1.57 (-1.87 to -1.28)	0.72 (0.65 to 0.79)	0.72 (0.65 to 0.80)	57.44 (39.71 to 83.08)
Surgery alone	0.32 (0.29 to 0.34)	-6.91 (-7.82 to -6.00)	-1.65 (-2.13 to -1.18)	0.86 (0.73 to 1.02)	0.63 (0.53 to 0.74)	10.77 (6.66 to 17.43)
Surgery and chemotherapy	0.23 (0.21 to 0.26)	-6.74 (-7.75 to -5.74)	-1.67 (-2.19 to -1.14)	0.70 (0.59 to 0.85)	0.63 (0.52 to 0.75)	21.81 (13.94 to 34.12)

